# Anti-diabetic drug utilization of pregnant diabetic women in us managed care

**DOI:** 10.1186/1471-2393-14-28

**Published:** 2014-01-17

**Authors:** Caitlin A Knox, Joseph AC Delaney, Almut G Winterstein

**Affiliations:** 1 Department of Pharmaceutical Outcomes & Policy, College of Pharmacy, Gainesville, FL, USA; 2 Department of Epidemiology, University of Washington, Seattle, WA, USA; 3 Colleges of Medicine and Public Health and Health Professions, University of Florida, Gainesville, FL, USA

**Keywords:** Pharmacoepidemiology, Drug utilization, Pregnancy, Managed care

## Abstract

**Background:**

With the increasing prevalence of type 2 diabetes in young adulthood, treatment of diabetes in pregnancy faces new challenges. Anti-diabetic drug utilization patterns of pregnant women with pre-existing diabetes are poorly described. We aim to describe anti-diabetic (AD) agent utilization among diabetic pregnant women.

**Methods:**

We utilized IMS LifeLink, including administrative claims data of patients in US managed care plans, to establish a retrospective cohort of women, age 18–46 years (N = 96,740) with billed procedures for a live birth, and a 12 month eligibility period before and 3 month after delivery. Diabetes mellitus was identified from ≥2 in- or outpatient claims with diagnoses (ICD-9-CM 250.XX) before pregnancy. We estimated the prevalence of AD drugs before, during and after pregnancy, and secular trends across the study period (1999–2009), using linear regression. A sensitivity analysis was conducted to identify the extent of misclassification of trimesters.

**Results:**

Almost six percent (n = 5,581) of the live birth cohort had diabetes mellitus. Throughout the study, 48% (1999) and 78% (2009) (p < 0.0001) of diabetic women received AD drugs during pregnancy. The most common AD drugs during pregnancy were insulin, metformin, sulfonylureas, thiazolidinediones (TZD), and combination AD. The annual prevalence of insulin use increased by only 1% from 39% (1999) to 40% (2009) (p = 0.589) during pregnancy, while use of sulfonylureas and metformin increased from 2.5% and 4.2% (1999) to 17.3% and 15.3% (2009) (p < 0.0001), respectively. Insulin and sulfonylurea use steadily increased in prevalence from the 1st to 3rd trimester (16.5% and 3.3% to 33.0% and 7.5%), while metformin and TZD use decreased (11.4% and 1.6% to 3.8% and 0.2%).

**Conclusions:**

AD use during pregnancy demonstrates the need for additional investigation regarding safety and efficacy of AD drugs on maternal outcomes.

## Background

Current estimates project that by 2025 one in three adults in the United States (US) will have diabetes mellitus [1]. In 2010, approximately 11% of US women aged 20 years or older were either diagnosed or had undiagnosed diabetes [1]. This reflects an increase in diabetes prevalence of 2% in this age group over the last five years, with a corresponding 1.9 million new cases of diabetes diagnosed in 2010 [1]. This growth is almost exclusively attributable to type 2 diabetes mellitus, which traditionally has had its onset in later stages of adulthood [1,2].

The growing prevalence of type 2 diabetes in young adults is particularly important, as more young women will be diagnosed during reproductive years [2]. Poorly controlled diabetes both before and during the first trimester of pregnancy can cause major birth defects, spontaneous abortions, and stillbirths [2]. Despite this well-established fact, more than 60% of women with pre-existing diabetes have difficulty managing their glycemic control during pregnancy [3-5]. Researchers and providers agree that glycemic control is one of the most important modifiable risk factors in minimizing birth defects of infants born to women with pre-existing diabetes [6-10]. However, little experience and evidence regarding the safety and effectiveness of oral agents during pregnancy exists.

While type 1 diabetes management requires insulin and thus leaves little choice during pregnancy, type 2 diabetes may be managed with life-style modifications, oral anti-diabetic agents, and/or insulin. Among oral agents, several new molecular entities have been added within the last ten years with limited data on pregnancy outcomes. Given the limited research that is available on anti-diabetic agent use during pregnancy, we aimed to describe anti-diabetic agent utilization before, during and after pregnancy and determine secular trends among classes of anti-diabetic drugs across the 10-year study period (1999–2009) in women with pre-existing diabetes.

## Methods

We utilized the IMS LifeLink Database, which consists of commercial health plan information from more than 100 managed care plans throughout the US. The majority of the payer type within the database is commercially insured. The IMS LifeLink database also includes Medicaid, Medicare, self-insured and unknown payer types. The database records are generally representative of the commercially insured population in terms of gender and age. The IMS LifeLink database is comprised of eligibility and demographic information, as well as, inpatient and outpatient claims data with detail on diagnosis and procedures, and prescription drug claims. This database contained a random sample of 6 million women aged 18 to 46 years with no prescription drug claim for contraceptives. To be included in our study cohort we required women to have a billed medical procedures code for live birth (Table 1), and 12 months continuous insurance coverage before and 3 months after delivery. Women were required to have at least one prescription drug claim before pregnancy, to confirm prescription drug coverage. A total of 96,740 women met the inclusion criteria for the cohort.

**Table 1:** Delivery-related procedure (CPT-4) codes used to identify live births

**Code**	**Description**
01960	Anesthesia for vaginal delivery only
01961	Anesthesia for cesarean delivery only
01962	Anesthesia for urgent hysterectomy following delivery
01963	Anesthesia for cesarean hysterectomy w/o any labor analgesia/anesthesia care
01967	Neuraxial labor analgesia/anesthesia, planned vaginal delivery
01968	Anesthesia for cesarean delivery following neuraxial labor analgesia/anesthesia
01969	Anesthesia for cesarean hysterectomy following neuraxial labor analgesia/anesthesia
59050	Fetal monitoring in labor, physician w/written report
59051	Fetal monitoring in labor, physician w/written report; interpretation only
59400	Routine obstetric care, antepartum care, vaginal delivery, & postpartum care
59409	Vaginal delivery only (w/wo episiotomy &/or forceps)
59410	Vaginal delivery only (w/wo episiotomy &/or forceps); w/postpartum care
59412	External cephalic version, w/wo tocolysis
59414	Delivery, placenta (separate procedure)
59430	Postpartum care only (separate procedure)
59510	Routine obstetric care w/antepartum care, cesarean delivery, & postpartum care
59514	Cesarean delivery only
59515	Cesarean delivery only; w/postpartum care
59525	Subtotal/total hysterectomy after cesarean delivery
59610	Routine obstetric care, vaginal delivery, w/antepartum, postpartum care, previous c-section
59612	Vaginal delivery only, previous cesarean delivery
59614	Vaginal delivery only, previous cesarean delivery; w/postpartum care
59618	Routine obstetric care, ante/postpartum, cesarean delivery after failed vaginal delivery, previous cesarean delivery
59620	Cesarean delivery, after failed vaginal delivery, previous cesarean delivery
59622	Cesarean delivery, after failed vaginal delivery, previous cesarean delivery; w/postpartum care
99436	Attendance at delivery, at request of delivering physician, & stabilization of newborn
99440	Newborn resuscitation

To identify patients with pre-existing diabetes, we required two in- or outpatient claims with diagnosis of diabetes mellitus (International Classification of Diseases, Ninth Revision, Clinical Modification (ICD-9-CM) 250.XX) within the 3 months preceding conception [11,12]. ICD-9-CM codes identify the specific type of diabetes mellitus by the fifth digit of the code, but previous research suggests that current coding practices do not provide sufficient accuracy to identify the type of diabetes from the fifth digit of the ICD-9-CM code [13,14]. Therefore, we did not separate women according to their diabetes mellitus type, but decided to present a subgroup analysis in women with high propensity to have type 2 diabetes [15]. For the analysis, we assumed the presence of type 2 diabetes when 100% of diabetes mellitus diagnoses in the claims indicated type 2 diabetes (ICD-9-CM 250. × 0 or 250. × 2) [15]. The “other diabetes” subgroup included all women with type 1 diabetes mellitus claims and those with mixed or unspecific claims (i.e., missing the 5th digit).

We defined the delivery date as the date of the first Current Procedural Terminology (CPT-4) claim for live birth for each woman. We included women with live births only because we needed to obtain an estimated conception date for each woman. Inclusion of terminated pregnancies would not have allowed the determination of trimesters because the date of termination relative to conception would be unknown. Since we did not have access to the Last Menstrual Period (LMP) date, it was necessary for us to calculate the conception date of the pregnancy. This was done by subtracting nine months (270 days) from the delivery date [16,17]. Each trimester of pregnancy was assigned a 91-day interval [16,17]. Pre-pregnancy interval were identified as the three months preceding the imputed conception date to classify the presence of pre-existing diabetes and capture pre-pregnancy drug utilization. We separately defined an after pregnancy interval as the three-month time period after the delivery date, resulting in five distinct 3-month periods, which were used to define drug use prevalence.

Because we relied on an imputed conception date, there was a potential for misclassification of the pre-pregnancy period and the following trimesters. The risk of misclassification of the pregnancy periods was particularly high in women with diabetes, because they are at higher risk for pre-term births (i.e. shorter gestation periods). In order to evaluate this effect we conducted a sensitivity analysis, where we utilized the first healthcare encounter with a pregnancy code (ICD-9-CM: V22, V23, V72.40, V72.42 and CPT-4:81025) to estimate conception date [18]. Using the first pregnancy claim as the conception date, the mean length of pregnancy for the cohort was 6.55 months with at standard deviation of 1.64 months. Five percent of the diabetes cohort had an ICD-9-CM claim for early delivery (644.2, 644.20, and 644.21). Therefore, we conducted another sensitivity analysis where we adjusted the gestation length to 245 days for women with an early delivery claim [19]. We saw that the drug class utilization in the sensitivity analysis did not differ significantly with the originally imputed pregnancy periods we calculated using 270 days subtracted from the delivery date.

Among the ten anti-diabetic drug classes identified within the IMS pregnancy cohort, we focused our analysis on the five most commonly utilized drug classes before, during and after pregnancy: insulin, biguanides, sulfonylureas, thiazolidinediones, and oral anti-diabetic combinations. The remaining anti-diabetic drug classes all showed prevalence rates of less than 1% (alpha-glucosidase inhibitors, amylin analogs, dipeptidyl peptidase-4, GLP-1 receptor agonists and meglitinide analogues). All insulin products were collapsed into one category reflecting American Hospital Formulary Service (AHFS) drug class 68.20.08. We calculated respective drug class prevalence as the proportion of diabetic women with a drug claim of a particular class during each of the designated periods.

We estimated the prevalence along with the 95% confidence intervals of anti-diabetic drug class utilization before, during (for each trimester) and after pregnancy. We investigated the secular trend of annual drug utilization prevalence using linear regression. All analyses were conducted with SAS 9.2, Cary, NC. The University of Florida Institutional Review and Privacy Boards approved this study.

## Results

Over the course of the ten-year study period (1999–2009), we identified 5,581 (5.9%) women with pre-existing diabetes among all women with a procedure code for live birth. A total of 4,043 women had only ICD-9-CM codes consistent with type 2 diabetes mellitus. Diabetic women were on average slightly older, had more physician visits and more prescription drug claims before pregnancy (Table 2). As expected, diabetic women had a higher prevalence of additional co-morbid conditions than non-diabetic women had, but had similar frequencies of smoking, alcohol and drug abuse. Diabetic women also had a higher prevalence of cesarean section delivery compared to non-diabetic women, with 44.8% versus 27.2% respectively. There was a higher prevalence of diabetics in the East region of the US, and a lower prevalence in the South and West. Within the sub-group analysis, type 2 diabetic women had similar baseline characteristics, but the average number of AD prescriptions before pregnancy was lower in the type 2 diabetes only group when compared to all diabetic women.

**Table 2:** Baseline characteristics of the study cohorts

**Variable**	**Category**	DM*	DM*		Not DM*
		**N = 5,581**	**N = 5,581**		**N = 91,159**
			T2DM*	Other DM*	
			**N = 4,043**	**N = 1,538**	
Age: Years (SD)		32.3 (5.1)	32.5 (5.1)	32.1 (5.0)	30.1 (5.3)
Eligibility: Months (SD)		56.8 (25.2)	57.4 (25.2)	55.1 (24.9)	51.3 (24.4)
Physician office visits 3 months before Pregnancy (SD)		21.3 (24.5)	20.8 (25.2)	22.5 (22.5)	14.6 (17.8)
Number of Prescription Drug Claims 3 months before Pregnancy (SD)	Anti-Diabetic	3.0 (7.5)	1.4 (4.5)	7.2 (11.2)	0.1 (0.9)
	Other	14.6 (18.8)	13.9 (18.5)	16.4 (19.4)	9.7 (12.4)
Delivery Route	Vaginal	55.2%	58.8%	47.7%	72.8%
	Cesarean Section	44.8%	41.2%	52.3%	27.2%
Comorbid Conditions	PCOS	12.5%	13.1%	10.9%	5.5%
	Hypertension	18.2%	17.0%	21.4%	4.3%
	Infertility	14.7%	15.6%	12.2%	10.6%
	IVF Claim	0.1%	0.1%	0.1%	0.0%
	Obesity	13.9%	13.4%	15.2%	3.8%
	Smoking	6.0%	6.0%	5.9%	4.6%
	Alcohol	0.8%	0.8%	1.0%	0.9%
	Drug Abuse	0.7%	0.7%	0.6%	0.7%
Region	East	32.6%	33.6%	26.6%	20.6%
	Midwest	43.1%	43.1%	40.6%	43.8%
	South	12.1%	11.1%	19.7%	17.9%
	West	12.3%	12.2%	13.2%	17.7%
Conception Year (SD)		2005 (2.4)	2005 (2.3)	2005 (2.6)	2005 (2.6)

*DM = pre-existing diabetes mellitus, T2DM = type 2 diabetes mellitus.

**Figure 1:**
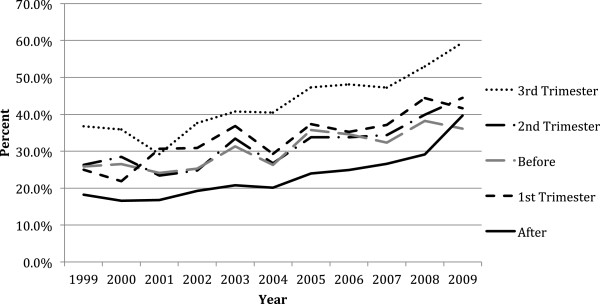
Annual prevalence of anti-diabetic treatment before, during and after pregnancy.

The annual mean age for the diabetic pregnancy cohort had a statistically significant increase from 29.85 years to 32.90 years from 1999 to 2009 (beta = 0.34, p <0.0001). The prevalence of anti-diabetic treatment (insulin or oral anti-diabetic drugs) among diabetic women before pregnancy increased from 25.9% (CI: 25.74, 26.06) to 36.1% (CI: 35.94, 36.26) from 1999 to 2009 (beta = 1.3%, p <0.0001) (Figure 1). During pregnancy, we observed a statistically significant rise in anti-diabetic drug treatment from 48.3% in 1999 to 77.9% in 2009 (beta = 3.4%, p < 0.0001). The growth in the prevalence of treatment was similar across trimesters with the largest growth in the third trimester, which saw a 23% increase in treatment from 36.8% (CI: 36.58, 37.01) in 1999 to 59.4% (CI: 59.18, 59.62) in 2009 (beta = 2.4%, p <0.0001). The prevalence of diabetic women who were treated with an anti-diabetic drug after pregnancy approximately doubled from 18.3% (CI: 18.11, 18.49) to 39.7% (CI: 39.51, 39.89) from 1999 to 2009 at a rate of 1.8% (CI: 1.14, 2.53) per year (p <0.0001) (Figure 1).

**Figure 2:**
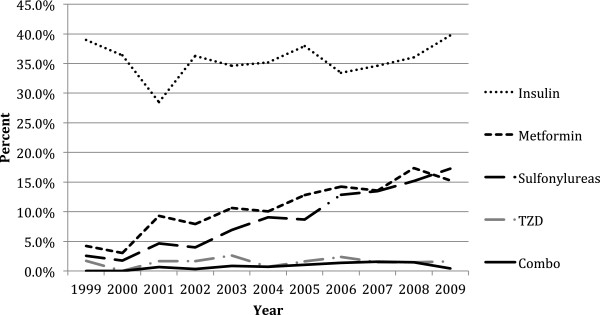
Annual prevalence of anti-diabetic drug utilization during pregnancy by drug class.

Among the various therapeutic classes, metformin and sulfonylureas showed the greatest increase over the study period from 4.2% (CI: 4.11, 4.29) and 2.5% (CI: 2.44, 2.56) in 1999 to 15.3% (CI: 15.21, 15.39) and 17.3% (CI: 17.26, 17.36) in 2009, respectively (p <0.0001 for each drug class) (Figure 2). The sulfonylurea drug class included glimepride, glipizide, and glyburide at 28%, 47% and 25% respectively. Thiazolidinediones and combination drugs remained constant at 1.6% (CI: 1.56, 1.64) and 0.5% (CI: 0.47, 0.53) over the course of the study. As expected rosiglitazone saw a decrease from 50% to 23.1% within the thiazolidinesdione drug class (beta = −3.4, p = 0.12). Insulin use during pregnancy increased 1% over the 10-year period from 39% (CI: 38.81, 39.19) in 1999 to 40% (CI: 39.81, 40.19) in 2009 (p-value = 0.589 (Figure 2).

**Figure 3:**
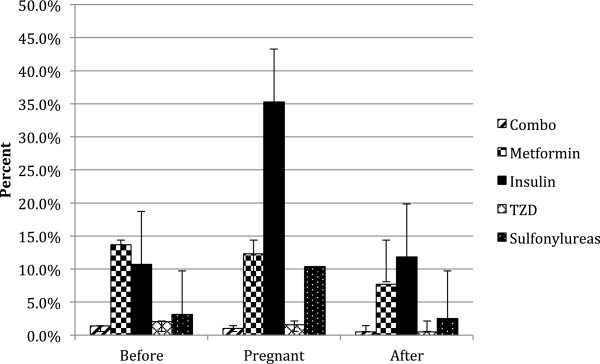
Pooled annual prevalence of anti-diabetic drug utilization, by drug class: before, during, and after pregnancy.

When pooled across the entire study period, the prevalence of metformin use before pregnancy in all diabetic women was 13.7% (CI: 13.11, 14.28), which decreased to 12.3% (CI: 12.21, 12.39) during pregnancy and then decreased further after pregnancy to 7.7% (CI: 7.62, 7.78) (Figure 3). Insulin utilization behaved as expected, with a lower average prevalence before pregnancy of 10.7% (CI: 10.52, 10.88), which more than tripled to 35.3% (CI: 35.11, 35.48) during pregnancy and then dropped again to 11.8% (CI: 11.67, 11.93) after pregnancy. Sulfonylurea utilization followed a surprisingly similar trend, as the pre-pregnancy baseline prevalence of 3.1% (CI: 3.08, 3.12) grew during pregnancy to 10.4% (CI: 10.34, 10.46) and then decreased to 2.5% (CI: 2.43, 2.57) after pregnancy.

In sub-group analysis of the type 2 diabetic women only, the pattern of anti-diabetic drug utilization was very similar to all diabetic women, except an expected lower prevalence of insulin use throughout each 3-month period. We noted an even lower rebound in utilization of metformin after pregnancy, with only 5.6% (CI: 5.51, 5.69) of type 2 diabetic women who used metformin post-delivery, but more than 12.4% (CI: 12.30, 12.50) before pregnancy.

## Discussion

We conducted a descriptive analysis on anti-diabetic drug utilization before, during and after pregnancy in diabetic women in order to guide future research on drug safety and effectiveness. Our study had several key findings. First, overall anti-diabetic drug utilization during pregnancy doubled over our ten-year study period. Second, despite this increase, we found a significant proportion of diabetic women with no drug treatment before, during and after pregnancy. Third, among the anti-diabetic drug classes we found interesting AD utilization pattern indicating changes in the treatment regimen during pregnancy. For example, the prevalence of metformin utilization decreased as pregnancy progressed from the 1st to the 3rd trimester, but was re-established into the treatment regimen after pregnancy, although at less than half the prevalence of the before period. Insulin and sulfonylurea demonstrated a reversed pattern, as the overall prevalence of utilization in both drug classes increased over the course of pregnancy and then returned to a low prevalence after pregnancy similar to utilization before.

Current recommendations for the initial therapy of diabetes (both type 1 and type 2) emphasize pharmacological interventions [20]. According to the American Diabetes Association (ADA) and the European Association for the Study of Diabetes (EASD), the initial management of type 2 diabetes should use combination therapy of metformin and lifestyle changes with augmentation of therapy with additional oral anti-diabetic drug to maintain glycemic control [20]. Or initiate insulin at diagnosis for individuals who present with severe hyperglycemic symptoms [20]. The recommendation reflects a change in treatment approaches a decade ago, which suggested that early type 2 diabetes might be managed with diet and lifestyle modifications alone. This evolution in treatment paradigm may explain the increase in overall anti-diabetic drug utilization that we observed among diabetic women before, during, and after pregnancy over our study period.

Interestingly, according to the National Health Interview Survey, for the past ten years the prevalence of diabetic adults who do not use insulin or oral anti-diabetic drugs to control their diabetes has slightly grown by 1% to more than 15% of US adults with diabetes [2,21]. The fact that we observed even higher proportions of non-treated patients in a managed care population with comprehensive drug benefits and increased medical attention due to pregnancy is surprising and raises questions about current treatment approaches. While the majority of pregnant woman may have had onset of type 2 diabetes in recent years and thus limited need for aggressive glycemic control, we still expected that current treatment guidelines and the emphasis on tight glucose control during pregnancy would have resulted in more comprehensive drug therapy. Unfortunately, because laboratory values are not available in claims data, it is unclear whether these women were able to achieve or maintain normoglycemia throughout pregnancy.

In general, the use of insulin to treat type 2 diabetes mellitus during pregnancy is accepted and recommended as safe and effective in achieving normal blood glucose levels [22,23]. There is also sufficient evidence to support that metformin is non-inferior to insulin with regards to neonatal safety, but comparative data regarding efficacy during pregnancy are lacking [24,25]. Furthermore, the majority of the metformin safety studies have been completed in women with polycystic ovarian syndrome or gestational diabetes, not in women with pre-existing diabetes, and have not evaluated pregnancy outcomes (pre-term labor, preeclampsia, cesarean section delivery) as safety endpoints [24,26].

The distinct increase of sulfonylureas use during pregnancy observed in this cohort was noteworthy in this context, because this drug class is currently not recommended for this indication, including an FDA contraindication for use in the last 4 weeks of pregnancy. This notwithstanding, several studies have found no harmful effects and report good glycemic control with the use of sulfonylureas during pregnancy [27-30]. Again, the majority of studies on the use of this drug class in pregnancy address the use in gestational diabetic women and lack evidence on various aspects of safety and efficacy.

We conducted this study using administrative claims data. The IMS LifeLink database allowed us to investigate the prevalence of diabetes in pregnancy in a relatively large cohort of more than 96,000 pregnant women, sampled from over 100 commercial health plans across the US, with fully adjudicated medical and pharmaceutical claims. The study period, which spanned ten years, allowed for the examination of time trends in drug utilization of these women before, during and after pregnancy. Focus on a managed care setting allowed good observation of prescribing practices, because access to care is not a major limitation.

All administrative data have limitations and this study is no exception. Despite our large sample size, focus on women in private insurance is not representative of diabetes treatment pattern in the US. In addition, the selection of health plans in IMS may not be representative as suggested by the geographic distribution of diabetes prevalence that does not follow nationally reported data. However, comparisons across time and across trimesters are expected to be valid within the study cohort. By restricting the pregnancy cohort to only women with a prescription drug claim before pregnancy, we are omitting women without drug coverage and theoretically omitting women who are not receiving drug treatment or who are non-adherent to their medication regimen. We potentially underestimated drug use prevalence in instances where pharmacy claims were not submitted for reimbursement because patients paid cash for their prescriptions. However, since prescription copays should have provided cheaper alternatives to patients during the study period, we expect minimal misclassification. We limited this descriptive analysis to pregnant women with live births. Therefore, the drug utilization patterns that were seen within this descriptive analysis may be different from pre-existing diabetic women with unsuccessful pregnancies (i.e. miscarriage, stillbirth, or termination).

Finally, we aimed to focus our study on patients with type 2 diabetes, but had to accept limitations in the granularity of ICD9-CM codes used for billing. Studies have been largely unsuccessful in validating algorithms to distinguish between diabetes types [13-15,31]. In order to estimate the extent of potential misclassification we provide results of a sensitivity analysis using a conservative approach that did not allow any codes for type 1 or unspecified diabetes in our sub-cohort of type 2 diabetic patients. This approach was likely not sensitive and may have excluded patients differentially.

## Conclusion

Pre-existing diabetes is an increasing comorbidity in pregnant woman in the US. While the overall use of anti-diabetic medications during pregnancy increased, a larger than expected proportion of pregnant women did not have AD drug claims throughout the study period. Contrary to current recommendations, metformin use decreased as pregnancy progressed while sulfonylurea use increased. The high rate of oral anti-diabetic drug use during pregnancy emphasizes the need for conclusive evidence regarding safety and efficacy in terms of glucose control as well as maternal outcomes. Further research is needed in order to evaluate the safety of oral anti-diabetic agent use in pregnant women with pre-existing diabetes in terms of pregnancy and neonatal outcomes. Within this study, it we were unable to assess the impact of glycemic control and co-morbid conditions on the choice of anti-diabetic agents throughout pregnancy. Therefore, it will also be important for future research to focus on the determinates of medication choices during pregnancy including the presence or on-set of co-morbid conditions and changes in glycemic control. Additionally, the lower prevalence of anti-diabetic drug utilization post-delivery indicates a possible need for further investigation.

## Competing interests

The authors declare that they have no competing interests.

## Authors’ contributions

Conceived and designed the research: CK, JD, AW. Analyzed the data: CK. Wrote the paper: CK. Interpretation of data: CK, JD, AW. Critically revised manuscript: CK, JD, AW. All authors have approved the manuscript as submitted.

## Presentation of work

The sub-group analysis of the type 2 diabetes data from this paper was presented at the 28th International Conference on Pharmacoepidemiology and Therapeutic Risk Management, in August 2012.

## Pre-publication history

The pre-publication history for this paper can be accessed here:

http://www.biomedcentral.com/1471-2393/14/28/prepub
